# Assessing the Willingness of Future Health Care Providers to Participate in Vaccine Trials: Cross-Sectional VACCELERATE European Study

**DOI:** 10.2196/84952

**Published:** 2026-10-09

**Authors:** Andreas Yiallouris, Charalampos Filippou, Christina Merakou, Christos D Argyropoulos, Sophia C Themistocleous, George Shiamakkides, Andreas Sarantopoulos, Ahmed Razi Shaikh, Evgenia Noula, Andria Nearchou, Elizabeth O Johnson, Maria Papaconstantinou-Leontidou, Charis Armeftis, George Astras, Fiona A Stewart, Kerstin Albus, Jon Salmanton-García, Janina Leckler, Anna Maria Azzini, Ruth Joanna Davis, Lenka Součková, Helena H Askling, Tobias Lindström Battle, Elena Álvarez-Barco, Augustina Mozeryte, Dimitrios Poulimeneas, Ioannis Kopsidas, Jana Baranda Prellezo, Olena Valdenmaiier, Stine F Jakobsen, Rebecca Jane Cox, Margot Hellemans, Greet Hendrickx, Pierre van Damme, Jordi Ochando, Theoklis E Zaoutis, Ligita Jancoriene, Patrick Mallon, Pontus Nauclér, Petr Husa, Evelina Tacconelli, Oliver A Cornely, Zoi-Dorothea Pana

**Affiliations:** 1 Department of Medicine, School of Medicine, European University Cyprus, Nicosia, Cyprus; 2 Department of Chemical Engineering, College of Chemicals and Materials, King Fahd University of Petroleum and Minerals, Dhahran, Saudi Arabia; 3 Institute of Translational Research, Cologne Excellence Cluster on Cellular Stress Responses in Aging-Associated Diseases (CECAD), Faculty of Medicine and University Hospital Cologne, University of Cologne, Cologne, Germany; 4 Department I of Internal Medicine, Center for Integrated Oncology Aachen Bonn Cologne Duesseldorf (CIO ABCD) and Excellence Center for Medical Mycology (ECMM), Faculty of Medicine, University Hospital Cologne, University of Cologne, Cologne, Germany; 5 Department of Diagnostic and Public Health, Infectious Diseases, University of Verona, Verona, Italy; 6 Department of Pharmacology, University Hospital Brno, Masaryk University, Brno, Czech Republic; 7 Department of Infectious Diseases, Karolinska University Hospital, Stockholm, Sweden; 8 Department of Global Public Health, Karolinska Institutet, Stockholm, Sweden; 9 Centre for Experimental Pathogen Host Research, School of Medicine, University College Dublin, Dublin, Ireland; 10 Department of Radiology, Nuclear Medicine and Medical Physics, Institute of Biomedical Sciences, Faculty of Medicine, Vilnius University Medical Faculty, Vilnius University, Vilnius, Lithuania; 11 Collaborative Center for Clinical Epidemiology and Outcomes Research (CLEO), Athens, Greece; 12 National Center of Microbiology, Instituto de Salud Carlos III, Madrid, Spain; 13 Department of Pharmaceutical Sciences and of Health, Microbiology Section, Faculty of Pharmacy, Universidad San Pablo CEU, Madrid, Spain; 14 Centre of Excellence for Health, Immunity and Infections, University of Copenhagen, Copenhagen, Denmark; 15 Department of Clinical Science, Influenza Centre, Bergen Energi (Norway), Bergen, Norway; 16 VAXINFECTIO, Centre of Evaluation of Vaccination, Faculty of Medicine and Health Science, University of Antwerp, Antwerp, Belgium; 17 Partner Site Bonn Cologne, German Center for Infection Research, Cologne, Germany; 18 Clinical Trials Centre Cologne (ZKS Köln), Faculty of Medicine and University Hospital Cologne, University of Cologne, Cologne, Germany; 19 Department of Basic and Clinical Sciences, School of Medicine, University of Nicosia, 6 Makedonitissas Avenue, Nicosia, CY-2417, Cyprus, 357 22 841500

**Keywords:** vaccine trials, clinical trial participation, health professional students, medical students, vaccine confidence, pandemic preparedness, VACCELERATE, Europe

## Abstract

**Background:**

Future health professionals are expected to recommend vaccines, communicate with patients about vaccine safety, and support clinical trial literacy and recruitment during public health emergencies. However, their own willingness to participate in vaccine trials, and the barriers that shape that willingness, remain incompletely characterized across Europe.

**Objective:**

This study aimed to assess willingness to participate in future vaccine trials among European students in health-related disciplines and to describe their perceived barriers, motivations, vaccine attitudes, and preferred sources of health information.

**Methods:**

We conducted an open, multilingual, cross-sectional web survey within the VACCELERATE network from June to November 2023. National coordinators distributed the survey through institutional mailing lists, student associations, and program channels in participating European countries. Eligible participants were students aged 18 years or older enrolled in medicine, dentistry, nursing, pharmacy, psychology, biomedical sciences, physiotherapy, occupational therapy, or other health-related programs. The questionnaire assessed demographic characteristics, primary sources of health information, vaccine attitudes, willingness to participate in future vaccine trials, barriers among unwilling or unsure respondents, and motivations for participation. Analyses were descriptive, and denominators varied by item because nonmandatory questions could be skipped.

**Results:**

Of 1381 eligible respondents, 1192 (86.3%) answered the primary willingness question: 32.9% (n=392) were willing to participate in a future vaccine trial, 38.8% (n=463) were unsure, and 28.3% (n=337) were unwilling. Among the 769 respondents who were unwilling or unsure and selected barriers, the most frequent concerns were potential side effects (n=574, 74.6%) and not wanting to be a “test subject” (n=276, 35.9%). Among 1289 respondents included in the motivation analysis, commonly selected motivators included knowing the risks and side effects involved (n=575, 44.6%), becoming part of the solution to an important health problem (n=584, 45.3%), and knowing that personal safety would be protected (n=500, 38.8%).

**Conclusions:**

In this multicountry European sample of health professional students, only one-third reported willingness to participate in future vaccine trials, while the largest group was unsure. The findings suggest that trial-readiness initiatives for future health professionals should prioritize transparent communication about safety monitoring, risks and side effects, participant protections, and the practical conduct of vaccine trials. Because recruitment used an open convenience approach, the results should be interpreted as exploratory and hypothesis generating rather than representative of all European health professional students.

## Introduction

The COVID-19 pandemic demonstrated that vaccine development alone is insufficient for an effective public health response. Vaccine confidence, transparent communication, and public willingness to participate in vaccine research are also essential components of epidemic and pandemic preparedness [1-4]. In this context, health care professionals (HCPs) occupy a central position because they translate scientific evidence into patient-facing recommendations and often serve as trusted sources of vaccine information.

Health professional students are a particularly relevant group for vaccine research readiness. Although they are not the only potential clinical-trial participants, they are future clinicians, pharmacists, dentists, nurses, psychologists, biomedical scientists, physiotherapists, and allied health professionals who may later recommend vaccines, counsel vaccine-hesitant patients, explain clinical-trial safeguards, and support recruitment to ethically conducted studies. Previous studies among health care students and young adults suggest that vaccine attitudes and willingness to participate in trials are shaped by knowledge, trust, perceived safety, prior vaccination behavior, and information sources [1,2,5-8]. Understanding this group can therefore inform both curriculum development and future trial-engagement strategies.

In Europe, VACCELERATE was established in 2021 to strengthen coordination and infrastructure for vaccine trials and to support preparedness beyond the acute COVID-19 pandemic emergency [4,9-16]. A research-ready workforce requires not only technical capacity but also future HCPs who understand vaccine-trial design, participant protections, safety oversight, and the ethical basis of voluntary participation.

Against this background, this VACCELERATE survey aimed to assess willingness among students in health-related disciplines across multiple European countries to participate in future vaccine trials. Secondary aims were to describe barriers among students who were unsure or unwilling, motivations among potential participants, vaccine attitudes, and preferred sources of health information. COVID-19 pandemic–specific willingness, pandemic-related attitude change, previous trial participation, and subgroup analyses were treated as secondary descriptive context and are reported mainly in the supplementary material.

## Methods

### Study Design and Reporting Guidelines

We conducted an open, multilingual, cross-sectional web survey between June and November 2023 among university students enrolled in health-related disciplines in participating European countries. The study is reported in accordance with relevant elements of the CHERRIES (Checklist for Reporting Results of Internet E-Surveys) checklist for web-based surveys and the STROBE (Strengthening the Reporting of Observational Studies in Epidemiology) guidance for cross-sectional studies [17,18].

### Questionnaire Development

The questionnaire was developed specifically for the VACCELERATE survey to address the study objectives and included sections on demographics, health and COVID-19 vaccination status, previous clinical-trial participation, willingness to participate in COVID-19 and non–COVID-19 vaccine trials, perceived barriers and motivations, willingness to participate in nonvaccine clinical trials, and sources of health information. The questionnaire was piloted for clarity before dissemination but was not designed or validated as a psychometric instrument. The full English-language questionnaire is provided in Multimedia Appendix 1.

### Participants and Eligibility

Eligible participants were students aged 18 years or older who were enrolled in medicine, dentistry, nursing or midwifery, pharmacy, psychology, biomedical sciences, physiotherapy, occupational therapy, or other health-related programs at participating institutions. Dentistry students were included because dental professionals contribute to prevention-oriented health counseling. The survey landing page described the study aims, voluntary nature of participation, data use, anonymity, and confidentiality. Proceeding beyond the landing page constituted electronic informed consent. Respondents could skip nonmandatory items and withdraw before submission.

### Survey Administration and Data Handling

The survey was open and hosted in Google Forms. No IP addresses, cookies, or direct identifiers were collected. Duplicate submissions were discouraged in the instructions and screened through time-stamp patterns and implausible repeated entries; no duplicates were flagged for exclusion. Not all items were mandatory, and item-level denominators therefore varied across analyses. The multiselect barrier and motivation items instructed respondents to select up to 3 options; however, the form did not technically enforce this limit. Responses exceeding 3 selections were excluded from the corresponding item-level analyses and this is acknowledged as a limitation. Data were exported to CSV and stored on secure institutional drives in accordance with applicable General Data Protection Regulation (GDPR) requirements.

### Outcomes and Variables

The primary outcome was self-reported willingness to participate in a future vaccine trial, assessed using the questionnaire item asking whether respondents would be interested in enrolling in a vaccine trial unrelated to COVID-19 pandemic (eg, for another infectious disease such as malaria, tuberculosis, or human papillomavirus). Responses were categorized as willing, unsure, or unwilling. Secondary outcomes included barriers among respondents who were unsure or unwilling, motivations for vaccine-trial participation, primary sources of health information, general vaccine attitudes, COVID-19 vaccine attitudes, previous participation in trials, and willingness to participate in COVID-19 vaccine and nonvaccine clinical trials. Health information sources were included because they may identify channels through which future trial-literacy interventions could be delivered; the questionnaire did not include formal coursework or curriculum as a separate option, which is noted as a limitation.

### Statistical Analysis

Categorical variables were summarized using frequencies and percentages. For each questionnaire item, percentages were calculated using the number of respondents who answered that specific item as the denominator. Because not all questionnaire items were mandatory, denominators varied between analyses.

The primary willingness outcome was presented using 3 response categories: willing, unsure, and unwilling. Respondents who selected “unsure” or “unwilling” were considered together only for the descriptive presentation of reported barriers.

Subgroup analyses by discipline, medical training stage, country of birth, and country of training region were descriptive. Missing responses were handled using item-level available-case analysis. Analyses were conducted using R (version 4.x; R Foundation for Statistical Computing) and Stata (version 17; StataCorp).

### Ethical Considerations

The study questionnaire received initial approval from the Cyprus National Bioethics Committee (EEBK ΕΠ 2021.01.118). The approval documentation was shared with participating countries, and additional local ethics approvals were obtained where required. Before accessing the questionnaire, potential participants were presented with an electronic study-information page describing the purpose of the study, eligibility criteria, voluntary nature of participation, intended use of the data, anonymity, confidentiality, and the right to withdraw before submitting the questionnaire. Participants were required to confirm their consent electronically before proceeding to the survey.

No names, IP addresses, cookies, or other direct personal identifiers were collected. Survey data were stored on secure institutional drives in accordance with applicable data protection requirements, including the GDPR. No financial compensation or other incentives were provided to participants.

### Missing and Item-Level Data

Not all participants answered all questions; therefore, denominators are reported separately for each item. Stratified analyses were descriptive and included discipline (medicine vs other health-related disciplines), medical training stage (preclinical medical students: years 1‐3, clinical medical students: years 4‐6), country of birth (European Union vs non–European Union), and country of training (northern European Union vs southern European Union). Participants reporting training in 2 countries contemporaneously (n=4) were excluded from the country of training analysis. Switzerland (n=1) and Norway (n=2) were also excluded from this stratification because they are not European Union member states. For vaccine-attitude items, “very positive” and “positive” were combined as “positive,” whereas “neutral,” “negative,” and “very negative” were combined as “neutral or negative.” For COVID-19 vaccine attitude, “very positive” and “positive” were combined as “positive,” whereas “neutral negative” and “very negative” were combined as “neutral or negative” to preserve cell sizes and improve interpretability. For self-reported health, the original response options were “very good,” “good,” “fair poor,” and “very poor.” For descriptive analysis, “very good” and “good” were combined as “good,” whereas “fair poor” and “very poor” were combined as “fair or poor.”

### Sample Size, Response Rate, Dissemination, and Recruitment

No formal a priori sample size calculation was performed because recruitment was based on a feasible, time-limited VACCELERATE network survey. The central study team prepared a standard survey invitation containing a brief description of the study, the eligibility criteria, and an open hyperlink to the online questionnaire. The invitation and survey link were circulated through the VACCELERATE network to national coordinators in participating European countries.

National coordinators facilitated dissemination within their respective countries through participating universities and other health professional training institutions. At the institutional level, the invitation was distributed through available institutional mailing lists, program- or course-level communication channels, student associations, and relevant student networks. Local institutional collaborators and, where applicable, student representatives supported the redistribution of the invitation to students enrolled in eligible health-related programs.

Recruitment followed a nonprobability convenience sampling approach. Participation was voluntary, and no financial or other incentives were provided. Because the questionnaire was accessed through an open link that could be redistributed within institutional and student networks, no centralized sampling frame was available. Therefore, the number of students who received or viewed the invitation could not be determined, and a formal response rate could not be calculated.

For the primary willingness item, 1192 respondents provided an answer. At this denominator, a proportion near 50% would have an approximate 95% precision of +2.8 to –2.8 percentage points, and the observed willing proportion of 32.9% would have an approximate precision of +2.7 to –2.7 percentage points. These calculations describe statistical precision only and do not overcome the potential selection bias inherent to convenience sampling.

## Results

### Participant Flow

A total of 1410 students submitted the online questionnaire. Twenty-six respondents were excluded because they did not meet eligibility prerequisites recorded in the screening questions, and 3 were excluded because they were no longer students. The final analytic sample therefore included 1381 eligible respondents. Because the survey was distributed through an open link, the denominator of all students who received or viewed the invitation could not be established.

### Country of Residence and Residential Setting

Respondents most resided in Germany (384/1381, 27.8%), Italy (189/1381, 13.7%), Cyprus (172/1381, 12.5%), the Czech Republic (170/1381, 12.3%), Sweden (167/1381, 12.1%), and Austria (108/1381, 7.8%).

Among respondents who reported a residential setting, 72.6% (1000/1378) lived in urban areas, 15.5% (214/1378) in semiurban areas, and 12% (164/1378) in rural areas (Table S1 in Multimedia Appendix 2). These results describe the responding sample and should not be interpreted as a representative distribution of European health professional students.

### Age, Gender, Health Status, Discipline, and Year of Training

Among the 1373 respondents who reported age, most were aged 18 to 24 years (n=987, 71.9%), followed by 25 to 34 years (n=331, 24.1%), 35 to 64 years (n=54, 3.9%), and 65 years or older (n=1, 0.1%). The single respondent aged 65 years or older was retained because student status and eligibility were confirmed through the survey responses. Among 1379 respondents who reported gender, 68.8% (n=949) were female, 29.7% (n=409) were male, 0.8% (n=11) identified as nonbinary or other, and 0.7% (n=10) preferred not to disclose. Most respondents were medical students (920/1305, 70.5%), the remaining respondents were enrolled in nursing or midwifery, psychology, pharmacy, dentistry, biomedical sciences, physiotherapy, or occupational therapy (Table 1).

**Table 1.** Demographic characteristics, self-reported health status, discipline, and year of training among eligible respondents in a cross-sectional VACCELERATE web survey of European students in health-related disciplines (June-November 2023).

Demographics	Participants, n (%)	
Age group (n=1373; years)		
18 to 24	987 (71.9)	
25 to 34	331 (24.1)	
35 to 64	54 (3.9)	
>65	1 (0.1)	
Gender (n=1379)		
Female	949 (68.8)	
Male	409 (29.7)	
Nonbinary or other	11 (0.8)	
Prefer not to say	10 (0.7)	
Health status (n=1380)		
Good	1323 (95.9)	
Fair or poor	57 (4.1)	
School of training (n=1305)		
Medicine	920 (70.5)	
Nursing (midwives)	153 (11.7)	
Psychology	49 (3.8)	
Pharmacy	47 (3.6)	
Dentistry	46 (3.5)	
Health sciences (biomedical sciences)	41 (3.1)	
Physiotherapy	29 (2.2)	
Occupational therapy	20 (1.5)	
Year of training (n=1318)		
First	338 (25.6)	
Second	313 (23.7)	
Third	238 (18.1)	
Fourth	170 (12.9)	
Fifth	162 (12.3)	
Sixth	97 (7.4)	

Most respondents reported having good health (1323/1380, 95.9%), while only a few (57/1380, 4.1%) reported having fair or poor health. The largest discipline groups after medicine were nursing or midwifery (153/1305, 11.7%) and psychology (49/1305, 3.8%). Regarding year of training, the largest proportions were first-year students (338/1318, 25.6%) and second-year students (313/1318, 23.7%).

### Primary Sources of Health Information

Health information sources were included to identify potential channels for future trial-literacy interventions. Among 942 respondents, the most commonly reported primary sources were health care websites, such as Mayo Clinic, Medscape, or WebMD (n=225, 23.9%); international health organization websites (n=169, 17.9%); health care providers, such as physicians or nurses (n=141, 15%); and independent online media sources, such as blogs or social media pages, groups, or accounts (n=140, 14.9%; Figure 1A). Traditional media refers to television, radio, and newspapers. The questionnaire did not offer coursework or curriculum as a separate response option, which limits interpretation among students in health-related disciplines.

**Figure 1.**
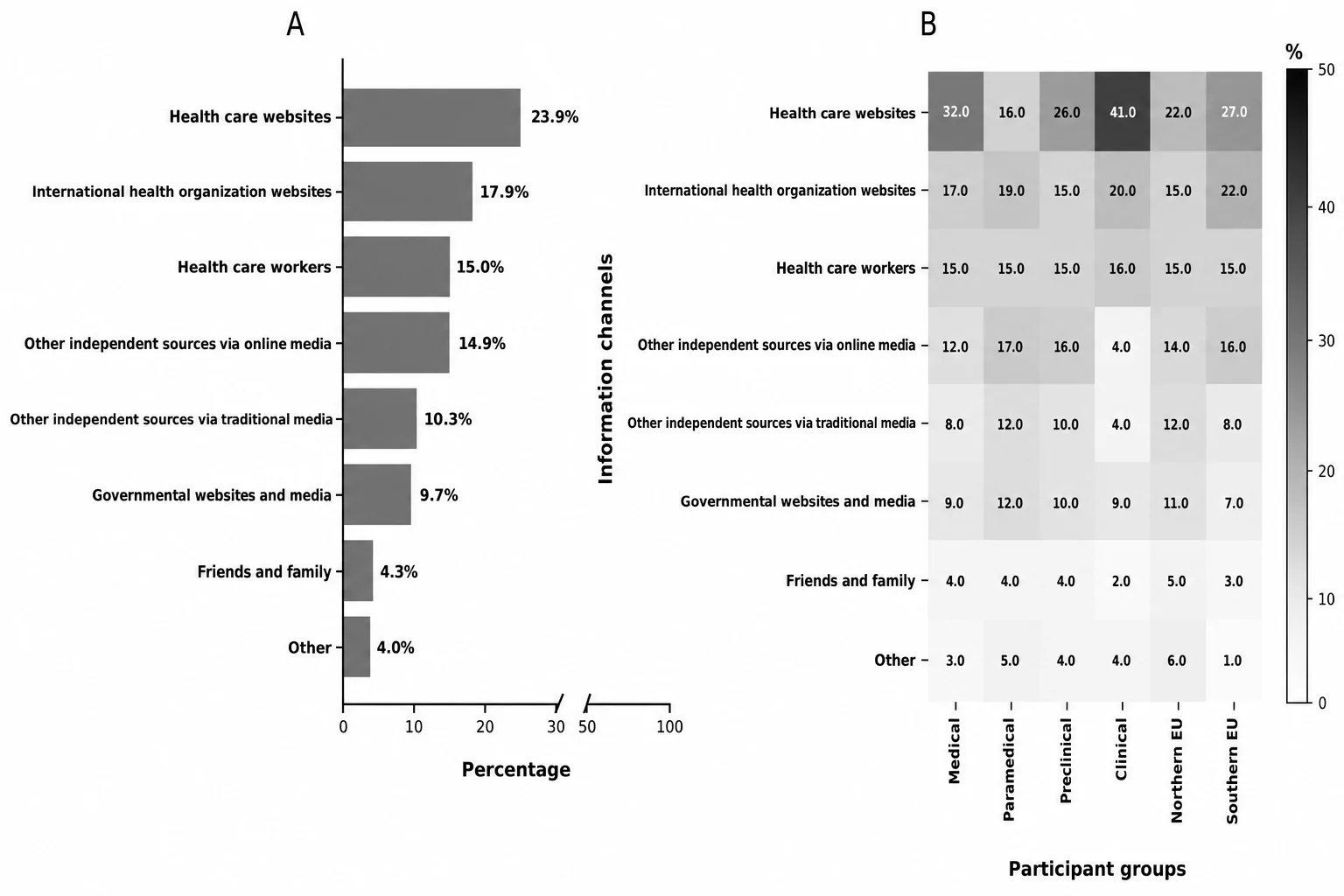
Primary sources of health information reported by respondents in a cross-sectional VACCELERATE web survey of European students in health-related disciplines, June-November 2023: (A) overall responses among students who answered the item (n=942); (B) descriptive heat map by subgroup (medical students, n=523; other health-related disciplines, n=345; preclinical medical students, n=317; clinical medical students, n=161; northern European Union (EU) training, n=533; southern EU training, n=396). Traditional media refers to television, radio, and newspapers.

Descriptive stratification by discipline, medical training stage, and country of training is shown in Figure 1B. The heat map is descriptive and was not designed for formal pairwise statistical comparisons across groups.

### General and COVID-19 Vaccine Attitudes

Overall, 90.1% of respondents reported a positive attitude toward vaccination in general, whereas 9.6% reported a neutral or negative attitude. For COVID-19 vaccination, 76% reported a positive attitude, 23.5% reported a neutral or negative attitude, and fewer than 1% preferred not to say (Figure 2).

Detailed subgroup analyses of vaccine attitudes by discipline, medical training stage, country of birth can be found in Multimedia Appendix 2 (Figures S1-S3) to keep the main text focused on the primary study objectives. COVID-19 pandemic–specific attitude-change results and previous trial participation results can be found in Multimedia Appendix 2 (Figures S4-S6), as secondary descriptive context.

**Figure 2.**
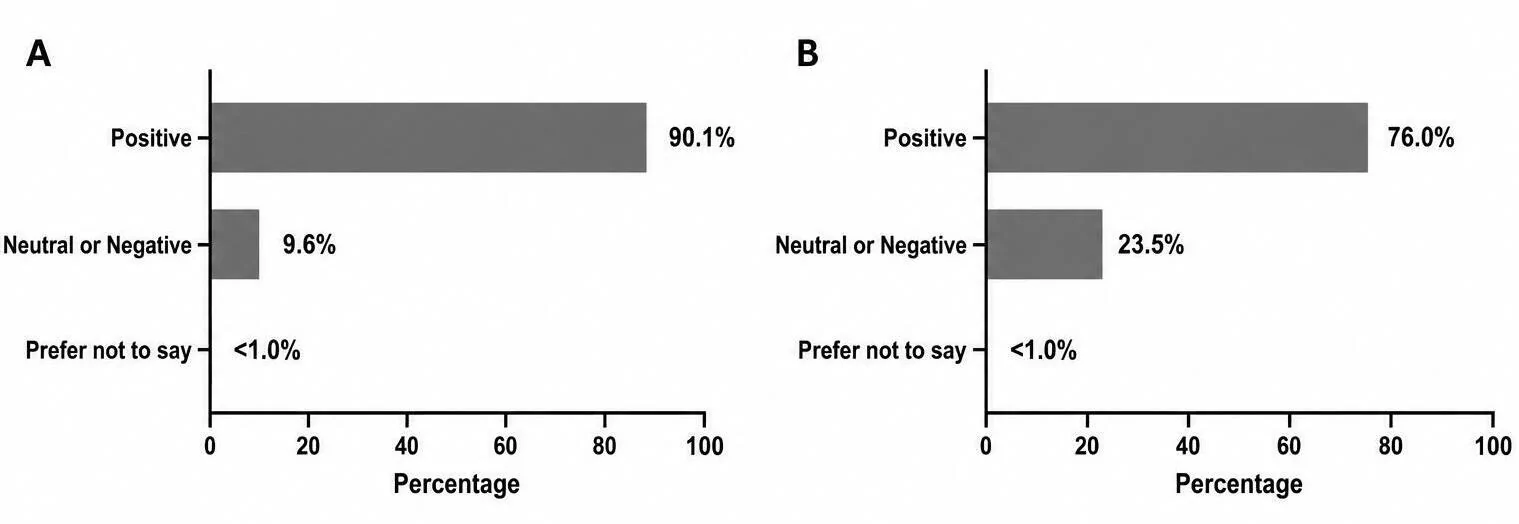
Attitudes toward (A) vaccination in general and (B) COVID-19 vaccination among respondents in a cross-sectional VACCELERATE web survey of European students in health-related disciplines, June-November 2023.

### Willingness to Participate in Future Vaccine Trials

Among 1192 respondents who answered the primary willingness question, the most common response was “I am not sure” (n=463, 38.8%), followed by “yes” (n=392, 32.9%), and “no” (n=337, 28.3%; Figure 3A).

**Figure 3.**
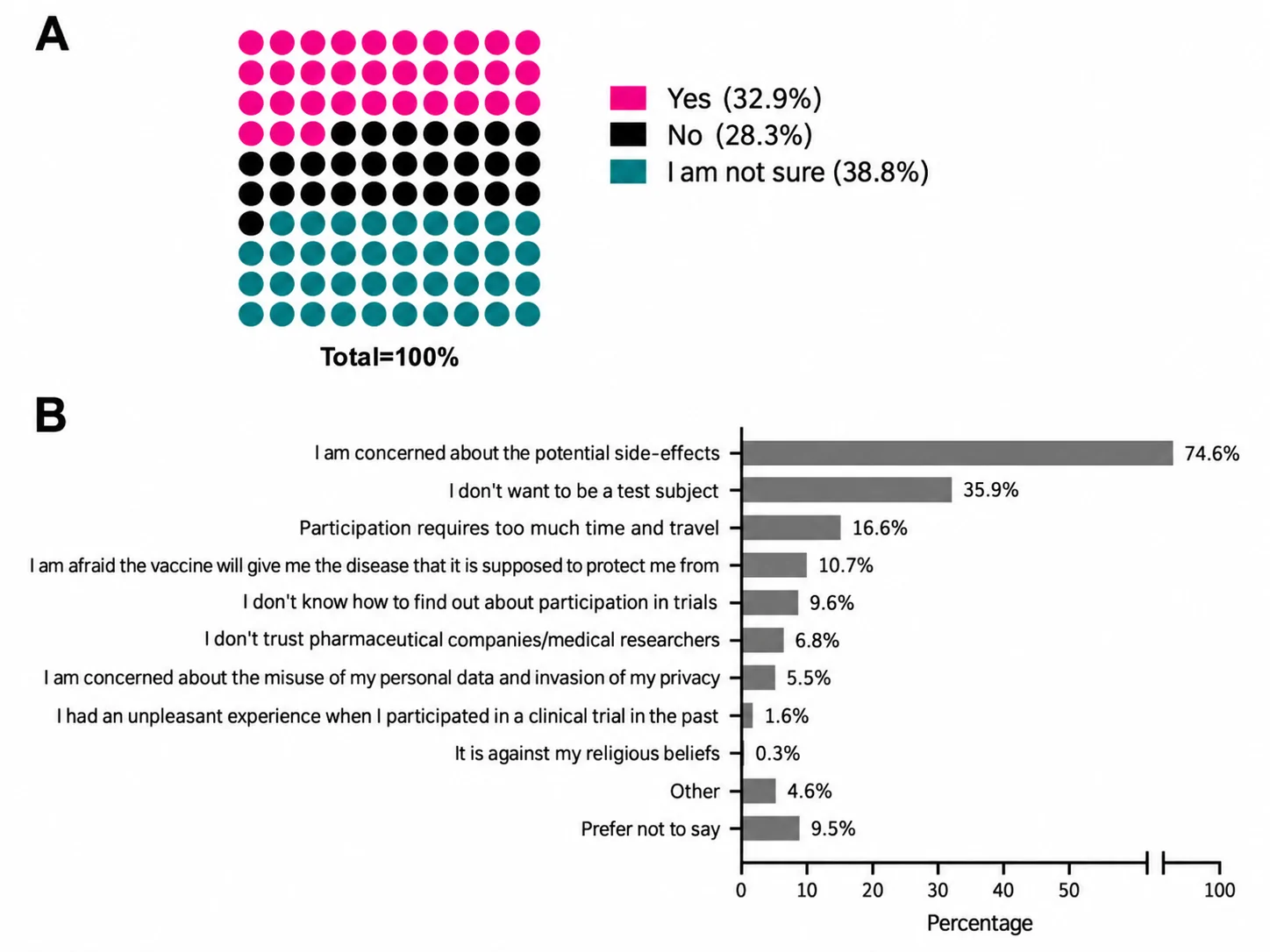
Willingness to participate in future vaccine trials and barriers among unwilling or unsure respondents in a cross-sectional VACCELERATE web survey of European students in health-related disciplines (June-November 2023): (A) primary willingness outcome among respondents who answered the item (n=1192) and (B) barriers among respondents who answered “no” or “I am not sure” and selected up to 3 barriers (n=769). Percentages in panel B use respondents, not selections, as the denominator and should not be summed.

Respondents who answered “no” or “I am not sure” were asked to select up to 3 barriers. Among 769 respondents included in this item-level analysis, the most frequently selected barriers were concern about potential side effects (n=574, 74.6%), not wanting to be a “test subject” (n=276, 35.9%), participation requiring too much time or travel (n=128, 16.6%), concern that the vaccine could cause the disease it is intended to prevent (n=82, 10.7%), and not knowing how to find trial-participation opportunities (n=74, 9.6%; Figure 3B). Because the survey form did not technically enforce the 3-option limit, 6 responses exceeding 3 selections were excluded from this analysis.

Subgroup analyses of willingness to participate in COVID-19 vaccine, other infectious-agent vaccine, and nonvaccine trials are presented in Figure S7 in Multimedia Appendix 2, rather than in the main text.

### Motivations to Participate in Future Vaccine Trials

Respondents could select up to 3 motivators for enrolling in a non–COVID-19 vaccine trial. Of 1378 responses received, 89 exceeded the selection limit and were excluded, leaving 1289 respondents in the item-level analysis. The most commonly selected motivators were knowing the risks and side effects involved (n=575, 44.6%), becoming part of the solution to an important health problem (n=584, 45.3%), knowing that personal safety would be protected (n=500, 38.8%), trusting those who designed the study (n=361, 28%), and receiving compensation for participation (n=341, 26.5%; Figure 4).

**Figure 4.**
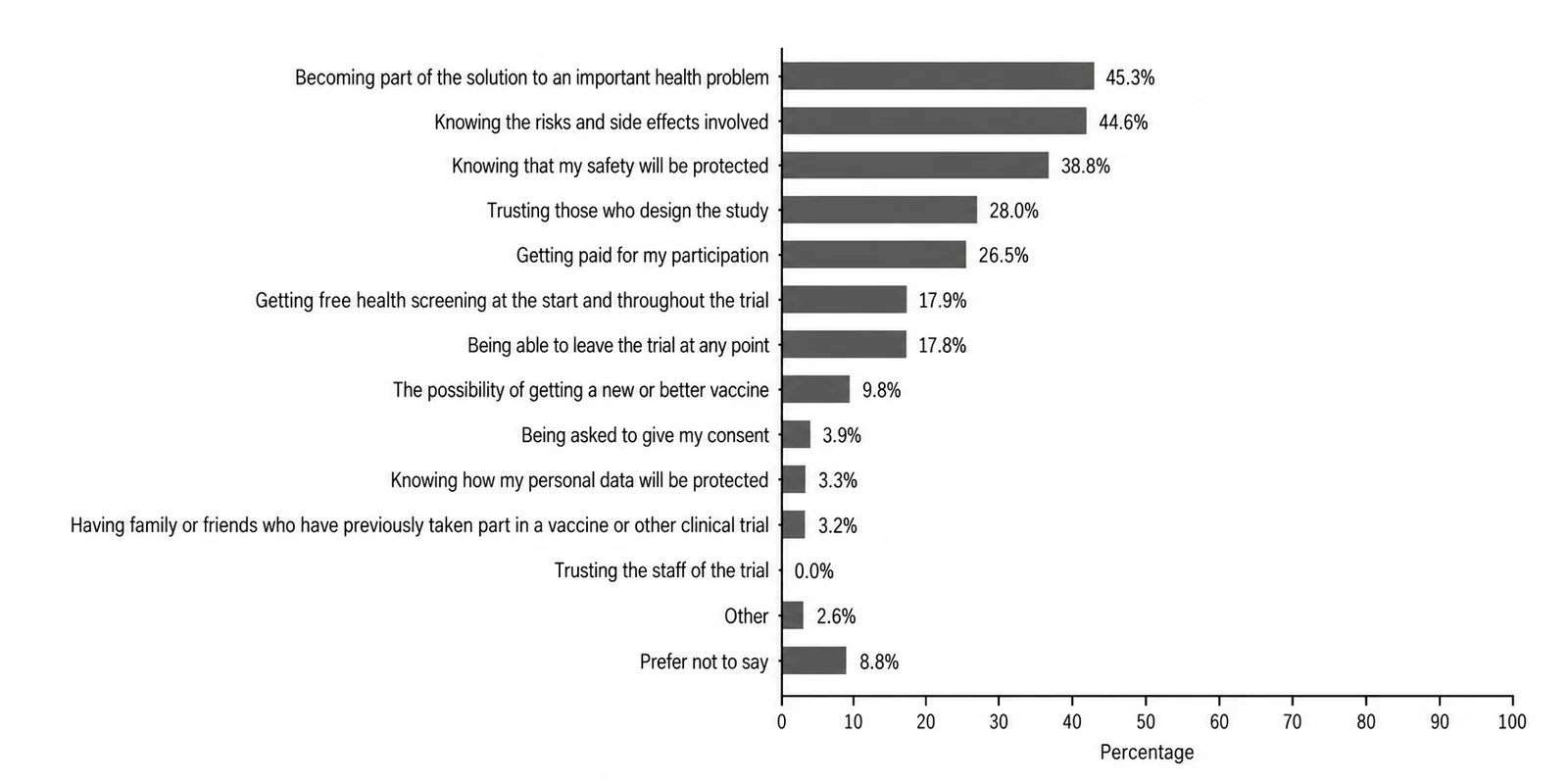
Motivations for potential participation in a future non–COVID-19 vaccine trial among respondents in a cross-sectional VACCELERATE web survey of European students in health-related disciplines (June-November 2023). Respondents could select up to 3 options; percentages use the number of respondents included in the item-level analysis (n=1289) as the denominator and should not be summed.

## Discussion

### Main Findings

This multicountry VACCELERATE survey found that willingness to participate in future vaccine trials among students in health-related disciplines was mixed: approximately one-third were willing, more than one-third were unsure, and more than one-quarter were unwilling. The predominance of an “unsure” group is important because it identifies a potentially reachable population for trial-literacy education, transparent communication about participant protections, and practical information about how vaccine trials are conducted.

The main barriers reported by unsure or unwilling respondents were concern about side effects and reluctance to be a “test subject.” These findings are notable because they arose in a population that is already training for health-related professions and reported generally favorable vaccine attitudes. They suggest that positive attitudes toward vaccination do not automatically translate into willingness to participate in vaccine trials. Educational approaches for future HCPs should therefore distinguish vaccine confidence from clinical-trial literacy, including safety monitoring, ethics review, informed consent, data protection, and the right to withdraw.

Motivators for potential participation also point toward modifiable recruitment and education targets. Respondents emphasized knowing the risks and side effects involved, knowing that their safety would be protected, and contributing to the solution of an important health problem. Recruitment materials and curricula that address both altruistic and self-protective motivations may therefore be more effective than messages focused only on public benefit.

These results align with prior studies showing that vaccine attitudes and trial willingness among students and health care workers are influenced by trust, safety concerns, knowledge, and information sources [1-3,5-8]. The present survey extends this literature by focusing on a multicountry European sample of future HCPs within a vaccine-trial preparedness network. Because students will later serve as patient counselors, role models, and sources of health information, their understanding of vaccine research may influence both future patient communication and trial recruitment.

The practical implication is that pandemic preparedness should include trial-literacy training for future HCPs, not only vaccine-knowledge training. Such training could address how trials are reviewed, how risks are monitored, how adverse events are managed, how personal data are protected, and how participants can find legitimate opportunities to enroll.

### Limitations

Several limitations should be considered. The survey used an open convenience sampling approach through the VACCELERATE national coordinators and institutional and student networks, so the number of students who received or viewed the invitation could not be determined, and a response rate could not be calculated. The sample should not be interpreted as representative of all European students in health-related disciplines. Selection bias is likely because students with stronger vaccine interest, stronger research interest, or greater trust in institutions may have been more likely to respond.

The data are self-reported and cross-sectional, which precludes causal inference. The questionnaire was developed for this VACCELERATE survey and piloted for clarity but was not a fully validated psychometric instrument. Some response categories were collapsed to improve interpretability and cell sizes, which may reduce granularity. The multiselect barrier and motivation items did not technically enforce the 3-option limit at data entry, leading to post hoc exclusion of responses exceeding the limit. In addition, the survey did not include coursework or curriculum as a specific health information source, and the June to November 2023 recruitment window provides a time-specific postpandemic snapshot rather than a current longitudinal assessment.

The barrier categories of “concern about potential side effects” and “not wanting to be a test subject” may overlap conceptually, and the present survey cannot determine whether they reflect distinct concerns or a shared underlying perception of risk. Qualitative follow-up through interviews or focus groups would be valuable to understand these concerns in greater depth and to design more precise educational interventions.

### Conclusions

This VACCELERATE survey suggests that many European students in health-related disciplines hold positive vaccine attitudes but remain uncertain about personal participation in future vaccine trials. Addressing concerns about side effects, participant protections, trial oversight, and practical trial logistics may help strengthen clinical-trial literacy among future HCPs and support a more research-ready health care workforce.

## Supplementary material

10.2196/84952 Multimedia Appendix 1 English-language VACCELERATE study questionnaire.

10.2196/84952 Multimedia Appendix 2 Supplementary figures and table presenting secondary and subgroup analyses.

## Abbreviations

CHERRIES: Checklist for Reporting Results of Internet E-Surveys
GDPR: General Data Protection Regulation
HCP: health care professional
STROBE: Strengthening the Reporting of Observational Studies in Epidemiology
