# Supplementary material for: Assessing the Willingness of Future Health Care Providers to Participate in Vaccine Trials: Cross-Sectional VACCELERATE European Study
Source: JMIR Public Health Surveill. 2026 Oct 9;12:e84952. doi: 10.2196/84952 (PMC13652525; doi:10.2196/84952)
Supplement: Supplementary file 1 [file publichealth-v12-e84952-s001.docx]

Multimedia Appendix 1

**English Questionnaire**

* Indicates required question

Where do you live right now? *

Shortly after the first outbreak of COVID-19, multiple vaccines were developed and licensed and have since played a significant role in fighting the pandemic. The development of all vaccines requires clinical trials, which rely on volunteer participation. Vaccine trials with humans can ensure that vaccines are non-toxic, safe, effective and, therefore, can offer protection against disease.

VACCELERATE is a network of research institutes that want to streamline vaccine development for COVID-19 and future outbreaks and to improve people's participation in vaccine trials in European countries. Through this survey, we aim to:

• Gain a better understanding of people's attitudes towards COVID-19 vaccine trials and vaccine trials in general.

• Identify what influences people's willingness to participate in vaccine trials of COVID-19 or other vaccine trials across different countries in Europe.

• Improve public health information and communication strategies regarding vaccine trials.

Why am I here? You are invited to participate in this anonymous survey which consists of 24 questions and will take approximately 10 minutes to complete. Please read the questions carefully and answer them as honestly as you can. There are no right or wrong answers. You do not need to create an account. You will not be asked for your contact information or any information that identifies you. Our questions are about your demographics (e.g. country of residence, age, gender), your state of health (e.g. high risk group, COVID-19), your experience with clinical trials and your willingness to participate in vaccine clinical trials. Your participation is voluntary. You can withdraw from the survey at any time by simply closing your browser window. None of your answers will be saved if you close your browser. However, if you reach the end of the questionnaire and submit it, your answers will be included in the study. After the final submission of the questionnaire, your answers will have been collected anonymously and you will no longer be able to make changes or withdraw your submission. If you have any questions regarding this study's design or conduct, please contact us at vaccelerate_cy@euc.ac.cy

If you agree to take part in the survey, please tick the boxes below: *

*Check all that apply.*

☐ I am 18 years old or older.

☐ I am on medical or paramedical training*.

☐ I have read and understood the purpose of the survey, how to participate, how to contact the study Coordinators and all other information provided.

☐ I agree that I wish to participate voluntarily in this survey and to have my answers used by the VACCELERATE consortium.

Please indicate your medical or paramedical training:

*Mark only one oval.*

○ School of Medicine

○ School of Nursing (Midwives)

○ School of Pharmacy

○ School of Physiotherapy

○ School of Occupational therapy

○ School of Psychology

○ School of Dentistry

○ School of Health Sciences (Biomedical Sciences)

Please indicate your year of medical/paramedical training *

*Check all that apply.*

☐ 1st year

☐ 2nd year

☐ 3rd year

☐ 4th year

☐ 5th year

☐ 6th year

A. Demographics

1. Country of birth *

____________________________________________________________________________

2. Country where your medical/nurse studies took place (if different than country of birth): *

____________________________________________________________________________

3. Age (in years) *

____________________________________________________________________________

4. Gender *

*Mark only one oval.*

○ Male

○ Female

○ Non-binary / Other

○ Prefer not to say

5. Area of residence *

*Mark only one oval.*

○ Urban (my city/town has a population of at least 50,000)

○ Semi-urban (my city/town has a population of at least 5,000)

○ Rural (not a city or town)

6. How many people do you share your household with, including yourself? *

____________________________________________________________________________

7a. Do you have children? *

*Mark only one oval.*

○ No

○ Yes

7.b. How many children do you have?

*Check all that apply.*

☐ 1

☐ 2

☐ 3

☐ 4

☐ 5

☐ 6+

7.c. Which age group/s does it/do they belong to? Select all that apply.

*Check all that apply.*

☐ Under 5

☐ 5-11

☐ 12-17

☐ 18+

8. What is your primary source of information about health topics? *

*Mark only one oval.*

○ International health organization websites

○ Health care websites (e.g., MayoClinic, MedScape, WebMD)

○ Governmental websites and media

○ Other independent sources via online media (e.g. blogs, social media pages/groups/accounts)

○ Other independent sources via traditional media (e.g. TV, radio, newspapers)

○ Healthcare workers (e.g. doctors, nurses)

○ Friends and family

○ Other, please clarify

8. Other, please clarify

____________________________________________________________________________

B. Health and COVID-19 vaccination status

9. How would you describe your health in general? *

*Mark only one oval.*

○ Very good

○ Good

○ Fair poor

○ Very poor

10. These are some conditions or characteristics of COVID-19 high-risk groups. Please check all that apply to you. *

*Check all that apply.*

☐ Over 60 years of age

☐ Smoker (current or former)

☐ Weakened immune system (immunocompromised)

☐ Dementia or other neurological condition

☐ Chronic kidney diseased

☐ Heart condition

☐ Diabetes (type 1 or type 2)

☐ Overweight or Obese

☐ Chronic liver disease

☐ Hypertension

☐ Stroke or cerebrovascular disease

☐ Cancer

☐ Chronic lung disease

☐ Mental health condition

☐ HIV infection or Tuberculosis

☐ Down syndrome

☐ Sickle cell disease or thalassemia

☐ Solid organ or blood stem cell transplant

☐ Substance use disorder

☐ Any other condition, please clarify

☐ None of the above

☐ Prefer not to say

11. What is your COVID-19 vaccination status? *

*Mark only one oval.*

○ Fully vaccinated, including booster shot(s)

○ Vaccinated with any licensed two-dose (AstraZeneca, Moderna, Pfizer BioNTech etc.) or single-dose vaccine (Johnson & Johnson), and at least 1 additional dose of any vaccine]

○ Fully vaccinated, without booster shot(s) [i.e., vaccinated with any licensed 2-dose (AstraZeneca, Moderna, Pfizer BioNTech etc.) or single-dose vaccine (Johnson & Johnson)]

○ Partially vaccinated (i.e. having received only one-dose of two-dose vaccines)

○ Not vaccinated

○ Prefer not to say

12. How would you describe your attitude towards vaccination, in general? *

*Mark only one oval.*

○ Very positive

○ Positive

○ Neutral

○ Negative

○ Very negative

○ Prefer not to say

13. How would you describe your attitude towards COVID-19 vaccination in particular? *

*Mark only one oval.*

○ Very positive

○ Positive

○ Neutral negative

○ Very negative

○ Prefer not to say

14. Could you say that your attitude towards vaccination changed after SARS-CoV-2 pandemic? *

*Mark only one oval.*

○ My attitude has been affected

○ My attitude has not been affected

○ I am not sure

○ Prefer not to say

15. Have you had COVID-19, confirmed by a PCR or rapid test? *

*Mark only one oval.*

○ Yes

○ No

○ Prefer not to say

C. Previous involvement in clinical trials.

The term "Clinical Trial" refers to a study with human participants that aims to help advance medical research. Clinical trials evaluate safety and effectiveness of new vaccines, new drugs, diagnostics or treatments.

16. Have you been a trial subject/participant? *

*Mark only one oval.*

○ Yes

○ No

○ Prefer not to say

17. Have you ever taken part in a trial assessing a vaccine (= vaccine trial)? *

*Mark only one oval.*

○ Yes

○ No

○ Prefer not to say

18. Have you ever taken part in a COVID-19 vaccine trial? *

*Mark only one oval.*

○ Yes

○ No

○ Prefer not to say

D. Willingness to participate in COVID-19 vaccine trials

19a. Would you be interested in enrolling as a subject/participant for a COVID-19 vaccine, in the future? *

*Mark only one oval.*

○ No — Skip to question 27

○ Yes — Skip to question 29

○ I am not sure — Skip to question 27

19b. Why not?

*Please select up to 3 reasons.*

*Check all that apply.*

☐ I had an unpleasant experience when I participated in a clinical trial in the past

☐ I am concerned about the potential side-effects

☐ I am afraid the vaccine will give me COVID-19

☐ I am concerned about the misuse of my personal data and invasion of my privacy

☐ I don't trust pharmaceutical companies

☐ I think it is unlikely that I will get COVID-19, because I am very healthy

☐ I don't trust medical researchers

☐ I don't need a COVID-19 vaccine, because I already had COVID-19

☐ I don't trust what the government says about COVID-19

☐ I don't think COVID-19 exists

☐ It is against my religious beliefs

☐ I don't want to be a test subject

☐ I don't know how to find out about participation in trials

☐ Prefer not to say

☐ Other, please clarify

19.b Other, please clarify

____________________________________________________________________________

20. If you were pregnant and/or breastfeeding, would you be interested in enrolling as a subject/participant for a COVID-19 vaccine ? *

*Mark only one oval.*

○ No

○ Yes

○ I am not sure

21. What would motivate you to enroll in a COVID-19 vaccine trial? *

*Please select up to 3 answers*

*Check all that apply.*

☐ Becoming part of the solution to an important health problem

☐ Knowing that my safety will be protected

☐ Knowing the risks and side-effects involved

☐ Getting paid for my participation

☐ Knowing how my personal data will be protected

☐ Being asked to give my consent

☐ Being able to leave the trial at any point

☐ Getting free health screening at the start and throughout the trial

☐ Being able to get to the hospital easily or cost-free

☐ Trusting the staff of the trial (e.g. doctors, nurses at the hospital)

☐ Trusting those who design the study (e.g. pharmaceutical company, university)

☐ The possibility of getting a new or better COVID-19 vaccine

☐ Having family or friends who have previously taken part in a vaccine or other clinical trial

☐ Prefer not to say

☐ Other, please clarify

21. Other, please clarify

____________________________________________________________________________

E. Willingness to participate in non-COVID-19 vaccine trials

22a. In the future, would you be interested in enrolling as a subject/participant in a vaccine trial that is not related to COVID-19 (e.g. for another infectious disease like malaria, tuberculosis, HPV)? *

*Mark only one oval.*

○ No

○ Yes

○ I am not sure

22b. Why? *

*Please select up to 3 reasons.*

*Check all that apply.*

☐ I had an unpleasant experience when I participated in a clinical trial in the past

☐ I am concerned about the potential side-effects

☐ I am afraid the vaccine will give me the disease that it is supposed to protect me from

☐ I am concerned about the misuse of my personal data and invasion of my privacy

☐ I don't trust pharmaceutical companies/medical researchers

☐ It is against my religious beliefs

☐ I don't want to be a test subject

☐ Participation requires too much time and travelling

☐ I don't know how to find out about participation in trials

☐ Prefer not to say

☐ Other, please clarify

22b. Other, please clarify

____________________________________________________________________________

23. What would motivate you to enroll in a non-COVID-19 vaccine trial? *

*Please select up to 3 answers.*

*Check all that apply.*

☐ Becoming part of the solution to an important health problem

☐ Knowing the risks and side-effects involved

☐ Knowing that my safety will be protected

☐ Getting paid for my participation

☐ Getting free health screening at the start and throughout the trial

☐ The possibility of getting a new or better vaccine

☐ Being asked to give my consent

☐ Knowing how my personal data will be protected

☐ Being able to leave the trial at any point

☐ Being able to get to the hospital easily or cost-free

☐ Trusting the staff of the trial (e.g. doctors, nurses at the hospital)

☐ Trusting those who design the study (e.g. pharmaceutical company, university)

☐ Having family or friends who have previously taken part in a vaccine or other clinical trial

☐ Prefer not to say

☐ Other, please clarify

23. Other, please clarify

____________________________________________________________________________

F. Willingness to participate in non-vaccine clinical trials

24. In the future, would you be interested in in enrolling as a subject/participant in a clinical trial that is not investigating a vaccine (for example, a clinical trial that is testing a new type of therapy, such as a drug)? *

*Mark only one oval.*

○ Yes

○ No

○ I'm not sure

G. Supplementary Question

25. How did you find out about this survey?

*Please select all that apply.*

*Check all that apply.*

☐ International health organisation websites and media (e.g. WHO, ECDC, CDC)

☐ Academic Institution (e.g., study network, professors)

☐ Governmental websites and media

☐ Other independent sources via online media (e.g. blogs, social media pages/groups/accounts)

☐ Other independent sources
