# Supplementary material for: Assessing the Willingness of Future Health Care Providers to Participate in Vaccine Trials: Cross-Sectional VACCELERATE European Study
Source: JMIR Public Health Surveill. 2026 Oct 9;12:e84952. doi: 10.2196/84952 (PMC13652525; doi:10.2196/84952)
Supplement: Supplementary file 2 [file publichealth-v12-e84952-s002.docx]

# **Multimedia appendix 2**

# **Supplementary Material**

# **Supplementary vaccine-attitude subgroup analyses**

**Students’ attitude towards vaccination by specific groups**

**Figure** S1. Attitudes toward general vaccination and COVID-19 vaccination by discipline among respondents in a cross-sectional VACCELERATE web survey of European students in health-related disciplines, June-November 2023.

The attitudes of students towards vaccination are analyzed and presented according to their discipline (Fig. S1). Among medical students, 96% expressed a positive attitude towards vaccination, compared to 78.7% of paramedical students (Fig. S1(a)-(b)). Regarding COVID-19 vaccination, 84.5% of medical students and 61.6% of paramedical students responded positively (Fig. S1 (c)-(d)).

**Figure** S2. Attitudes toward general vaccination and COVID-19 vaccination by medical training stage among respondents in a cross-sectional VACCELERATE web survey of European students in health-related disciplines, June-November 2023.

When examining the responses based on the year of medical training, 97.4% of medical students (clinical) and 94.8% of preclinical medical students showed a positive attitude towards vaccination (Fig. S2(a)-(b)). For COVID-19 vaccination, 87.9% of medical students (clinical) and 81.6% of preclinical medical students were positive (Fig. S2(c)-(d)).

**Figure** S3. Attitudes toward general vaccination and COVID-19 vaccination by country of birth among respondents in a cross-sectional VACCELERATE web survey of European students in health-related disciplines, June-November 2023. When analyzing by country of birth, 89.8% of EU-born students and 91.74% of non-EU-born students expressed positive attitudes towards vaccination (Fig. S3 (a)-(b)).

# **Supplementary COVID-19 attitude-change and previous-trial-participation analyses**

**Supplementary COVID-19 Pandemic Attitude-Change Analysis**

**Figure** S4. Self-reported change in general vaccination attitudes after the COVID-19 pandemic among respondents in a cross-sectional VACCELERATE web survey of European students in health-related disciplines, June-November 2023. Among the 1,381 student participants, when asked about whether their attitude towards vaccination had changed after the COVID-19 pandemic, 71.2% responded that their attitude had not been affected. In contrast, almost 18% indicated that their attitude had been affected, 10.07% were unsure, and 0.72% (10/1,381) preferred not to disclose their stance (Fig S4).

**Students’ previous participation in trials**

**Figure** S5. Previous trial participation among respondents in a cross-sectional VACCELERATE web survey of European students in health-related disciplines, June-November 2023: (a) participation in any clinical trial, (b) participation in any vaccine trial and (c) participation in a COVID-19 vaccine trial. When participants were asked if they had participated in any trials in the past, 83.1% responded with "no," 16% with "yes," and 0.72% preferred not to disclose their participation status (Fig. S5(a)). Regarding participation in vaccine trials, 3.05% indicated they had participated, 96.45% had not, and 0.51% chose not to say (Fig. S5(b)). Specifically, for COVID-19 vaccine trial participation, 2.82% answered "yes," 96.74% answered "no," and 0.43% (6/1,381) preferred not to disclose (Fig. S5(c)).

**Figure** S6. Previous trial participation by study discipline among respondents in a cross-sectional VACCELERATE web survey of European students in health-related disciplines, June-November 2023: (a-b) participation in any clinical trial, (c-d) participation in any vaccine trial and (e-f) participation in a COVID-19 vaccine trial, comparing medical and paramedical students. Fig. S6 summarizes the students’ previous trial participation by study discipline. Among the medical students, 18.05% reported past trial experience, 2.94% had experience with vaccine trials, and 2.61% specifically had experience with COVID-19 vaccine trials. Among the paramedical students, 13.51% indicated past trial experience, 3.65% had experience with vaccine trials, and 3.64% had specifically participated in COVID-19 vaccine trials.

# **Supplementary willingness subgroup analyses**

Willingness of students’ participation in future COVID-19 vaccine, other vaccine and non-vaccine trials by group.

**Figure S7.** Willingness to participate in future vaccine and non-vaccine trials by country-of-training region among respondents in a cross-sectional VACCELERATE web survey of European students in health-related disciplines, June-November 2023: willingness to participate in a COVID-19 vaccine trial among Northern EU-trained (a, n=962) and Southern EU-trained (b, n=401) students willingness to participate in a trial for a vaccine against another infectious agent among Northern EU-trained (c, n=962) and Southern EU-trained (d, n=212) students and willingness to participate in a non-vaccine clinical trial among Northern EU-trained (e, n=962) and Southern EU-trained (f, n=399) students. Within each panel, percentages sum to 100% and use respondents who answered that item as the denominator.

Table S1. Country of residence and residential setting among eligible respondents in a cross-sectional VACCELERATE web survey of European students in health-related disciplines, June-November 2023

| **Country of residence** | | |
| --- | --- | --- |
| **Country** | **Frequency (n)** | **Percentage (%)** |
| Austria | 108 | 7.82 |
| Cyprus | 172 | 12.45 |
| Czech Republic | 170 | 12.31 |
| Denmark | 9 | 0.65 |
| France | 2 | 0.14 |
| Germany | 384 | 27.81 |
| Greece | 22 | 1.59 |
| Ireland | 76 | 5.5 |
| Israel | 2 | 0.14 |
| Italy | 189 | 13.69 |
| Lithuania | 39 | 2.82 |
| New Zealand | 1 | 0.07 |
| Norway | 4 | 0.29 |
| Oman | 2 | 0.14 |
| Portugal | 1 | 0.07 |
| Slovakia | 8 | 0.58 |
| Spain | 20 | 1.45 |
| Sweden | 167 | 12.09 |
| Switzerland | 3 | 0.22 |
| The Netherlands | 2 | 0.14 |
| Total | 1381 | 100.00 |
| **Residence area** | | |
| Urban^1^ | 1000 | 72.57 |
| Semi-urban^2^ | 214 | 15.53 |
| Rural^3^ | 164 | 12 |
| Total | 1378 | 100.00 |

*Note.* ^1^ Urban (City/town with population of at least 50,000), ^2^ Semi-urban (City/town with population of at least 5,000), ^3^ Rural (not a city or town).
